# Persistent disparities in smoking among rural Appalachians: evidence from the Mountain Air Project

**DOI:** 10.1186/s12889-021-10334-6

**Published:** 2021-02-02

**Authors:** Kathryn Cardarelli, Susan Westneat, Madeline Dunfee, Beverly May, Nancy Schoenberg, Steven Browning

**Affiliations:** 1 College of Public Health, University of Kentucky, Lexington, KY USA; 2 Center for Health Equity Transformation, University of Kentucky, Lexington, KY USA; 3 College of Medicine, University of Kentucky, Lexington, KY USA

**Keywords:** Smoking, Tobacco, Appalachia, Health inequities, Disparities, Rural health, Respiratory health

## Abstract

**Background:**

Adult smoking prevalence in Central Appalachia is the highest in the United States, yet few epidemiologic studies describe the smoking behaviors of this population. Using a community-based approach, the Mountain Air Project (MAP) recruited the largest adult cohort from Central Appalachia, allowing us to examine prevalence and patterns of smoking behavior.

**Methods:**

A cross-sectional epidemiologic study of 972 participants aged 21 years and older was undertaken 2015–2017, with a response rate of 82%. Prevalence ratios and 95% confidence intervals for current smoking (compared to nonsmokers) were computed for the entire cohort then stratified by multiple characteristics, including respiratory health. Adjusted prevalence ratios for current smoking versus not smoking were also computed.

**Results:**

MAP participants reported current smoking prevalence (33%) more than double the national adult smoking prevalence. Current smoking among participants with a reported diagnosis of chronic obstructive pulmonary disease and emphysema was 51.5 and 53.3%, respectively. Compared to participants age 65 years and older, those age 45 years or younger reported double the prevalence of smoking (PR: 2.04, 95% CI: 1.51–2.74). Adjusted analyses identified younger age, lower education, unmet financial need, and depression to be significantly associated with current smoking.

**Conclusions:**

Despite declining rates of smoking across the United States, smoking remains a persistent challenge in Central Appalachia, which continues to face marked disparities in education funding and tobacco control policies that have benefitted much of the rest of the nation. Compared with national data, our cohort demonstrated higher rates of smoking among younger populations and reported a greater intensity of cigarette use.

## Background

Smoking in the United States (US) has declined in recent decades. From 2005 to 2019, the prevalence of cigarette smoking among US adults fell from 21 to 14% [1, 2]. The decline in cigarette smoking has not been experienced uniformly across US communities; rather, smoking rates have declined faster in urban compared with rural areas [3]. In rural regions, 28.5% of adults report smoking cigarettes, compared with 25.1% of urban adults [4, 5]. These differences in smoking prevalence underscore the need to examine smoking prevalence and behaviors in rural communities. Rural residents in the US are more likely to smoke than non-rural residents [3]. Furthermore, rural residents demonstrate greater intensity of cigarette smoking [6] compared to urban residents. In Central Appalachia, a region plagued by multiple health inequities, smoking rates have remained high over the last several decades [7], yet a detailed epidemiologic description of smoking behavior in this population is missing.

Tobacco, both its production and use, holds a significant place in the Central Appalachian region. Formerly considered a mainstay of the economy nationally and in Appalachia, beginning in 1975, production of tobacco in the US fell dramatically from 1.9 billion pounds to 890 million pounds, following the 1998 Master Settlement Agreement. This settlement agreement between states attorneys general and cigarette manufacturers provided incentives for farmers to produce alternative crops [8]. Nearly 45,000 Kentucky farms produced tobacco, with an average of 5.7 acres for each farm, prior to the settlement. Currently, less than one tenth of those farms still grow tobacco, the vast majority of small farmers having accepted cash compensation to halt production through the 2004 Tobacco Transition Payment Program, commonly referred to as the “tobacco buyout” [9]. But throughout this transition, Kentucky has remained the nation’s second largest producer of tobacco, with production of 123 million pounds in 2019 [10]. Some have speculated that the significant role of tobacco in the economy has given rise to an acceptance and embracement of tobacco [7].

Central Appalachia, which includes Appalachian Kentucky, has the highest prevalence of adult smokers in the nation, 25.2% compared to 16% in the non-Appalachian US. Further analysis by the Appalachian Regional Commission found that 45% of Appalachian counties fall in the highest quintile of adult smoking prevalence in the nation [11]. While the rate of adult smoking in Appalachian Kentucky has declined, following national trends, this decline has been significantly smaller than non-Appalachian counties in Kentucky. Within this population, attaining less than high school education or GED was associated with two and a half times increased odds of adult smoking. Furthermore, household income less than $15,000 was associated with nearly double the odds of smoking [12].

Marked disparities can be found in Appalachian Kentucky in prevalence and mortality from chronic illnesses for which tobacco use plays a primary role. For example, while the age-adjusted prevalence for chronic obstructive pulmonary disease (COPD) among adults is 5.9% [13] in the US, research conducted in Appalachian Kentucky found 19.6% of adults aged 40 years and over met the criteria for Global Initiative for Chronic Obstructive Lung Disease (GOLD) defined pulmonary obstruction [14]. A large proportion of those with moderate or severe obstruction did not self-report a medical diagnosis of any respiratory disease, suggesting that while COPD is highly prevalent in the region, it is also underdiagnosed. Appalachian Kentucky also had the highest rate of mortality due to COPD in the nation: 78.8 per 100,000 population compared to 42 per 100,000 in the US as a whole during 2008–2014. Similarly, mortality for heart disease for the same period was disproportionate in Appalachian Kentucky at 254 per 100,000 compared to 175 per 100,000 nationally [11]. All-site cancer mortality rate was also highest in Appalachian Kentucky at 227 per 100,000 compared to 168 per 100,000 nationally [11]. Appalachian Kentucky also leads the nation in incidence and mortality for cancers of the lung and bronchus, with incidence of 107.3 per 100,000 compared to 58.15 per 100,000 for the US and mortality of 78.8 per 100,000 compared to 41 per 100,000 nationally [15].

Given this excess smoking-related morbidity and mortality and in response to community concerns about the high rates of respiratory disease and other illness, the Mountain Air Project (MAP) was launched in 2015. Additional information about the project can be found in May et al., 2019 [16]. Although the specific reasons why smoking is so pervasive have been explored in this region [17, 18]—including familial and overall cultural acceptance and historical economic reliance on tobacco--we aimed to update the existing scholarship using a large, community-based sample. Moreover, we aimed to focus our examination on the state (Kentucky) with the highest rates of smoking in the US [19]. Thus, the aims of the present study were to (1) describe the smoking behaviors of this large adult Appalachian population, and (2) identify correlates of smoking in this population. To our knowledge, the Mountain Air Project represents the largest community-based cohort of adults in Appalachia examined for smoking behavior with this level of detail.

## Methods

### Study area and population

This study was conducted in two economically distressed rural counties [11], Harlan and Letcher, in Central Appalachia in southeastern Kentucky with a long history of health disparities, including the nation’s highest respiratory disease burden. According to the US Department of Agriculture, both counties are considered rural with rural-urban commuting area codes 7–9, with 10 designating the most rural commuting area [20]. The counties were selected based on the presence of underground coal and surface mining activities, documented community concerns regarding the health impacts of mining, high rates of respiratory disease, and the community infrastructure for mobilizing the project. The study was approved by the Institutional Review Committee of the University of Kentucky, and written informed consent was obtained from all participants in the study.

### Eligibility criteria and enrollment of participants

The study inclusion criteria included being an adult (aged 21+ years) male or female residing within a household in either of the two target counties, being an English speaker, and being of any race or ethnicity. Eligible households consisted of single-family residences, apartments or mobile homes. One adult was recruited per household. If an adult in the household reported having asthma, COPD, black lung disease, lung cancer, or other respiratory health condition, then the priority was to recruit that person for the study interview. If that person declined to participate and another adult household member without a respiratory condition was eligible, that person was recruited for the study. Participants received $40 for survey completion.

### Geographic site and household selection

The design of the study is a cross-sectional population survey and was conducted from November 2015 to August 2017. We used a stratified cluster sampling technique to randomly select small geographic areas in Harlan and Letcher counties for the sampling units. Community stakeholders suggested using “hollows” as the most relevant community geographic unit in defining “neighborhoods” for the epidemiologic survey. Hollows are watersheds of varying length, that are a common feature of the Appalachian landscape and vary widely in population, from no human habitation to communities with several hundred residences [16]. We defined candidate hollows using GIS map layers representing the boundaries of 14-digit hydrologic unit codes (HUCs). These are the smallest hydrologic units available, and often coincide with residential development patterns in the study region, since streets and homes are often ordered in linear fashion along narrow valleys. We obtained the GIS data for these HUCs from the Kentucky Geological Survey. We imported the HUC boundary polygons into ArcGIS 10.3 [21] and characterized the HUCs by their relationship to several other layers that characterized potential exposures to mining sites, roads and highways, and active oil and gas wells. Our final determination of the 40 hollows (HUCs) for sampling was based upon consideration of safety and accessibility of residences in these locations, along with community members’ guidance regarding the location of other mining-related facilities or hazardous manufacturing or waste sites such as powerplants, coal impoundment dams, processing facilities, or landfills.

Homes within the hollows were enumerated by field staff, with residences by hollow found to range from 0 to 397 residences. Within each hollow, homes were sampled by dividing the total number of homes in the hollow by an appropriate number to yield at least 10 homes per hollow for the study. Eligible homes were then selected by a systematic sample of every nth home using a random number generator to identify the first home. Due to the low numbers of residents in some of the HUCs, randomly selected replacement HUCs were provided to field staff to supplement study enrollments.

### Survey content, training, and administration

Community health workers (CHWs), most with previous experience in community-based research and familiarity with the local community, recruited and interviewed study participants. One CHW was responsible for determining eligibility of the household and recruiting. If an adult was willing to participate, the CHW obtained informed consent, collected demographic information and respiratory health status for each member of the household, and recorded the location of each home using the Global Positioning System. Each consenting participant was then assigned to a CHW who administered a questionnaire and collected spirometry. CHWs used REDCap survey software on iPads for all data collection [22]. Through REDCap, edit checks were programmed for automatic implementation as data were entered. Illogical or out-of-range values were flagged and interviewers were prompted to confirm data entered. Data entry accommodated inherent skip patterns. Details of the field operations for the MAP study are described elsewhere [16].

The survey, which took approximately 40 min for the CHWs to verbally administer, included questions to characterize participants’ baseline levels of established and potential risk factors for respiratory health outcomes, current and past symptoms of respiratory health over the past 2 and 12 months, and other behavioral and environmental questions. Questions for health outcomes were drawn primarily from established questionnaires, including the ISAAC questionnaire on wheezing and asthma, the Medical Research Council symptom-based questionnaire, and the Seattle Healthy Homes I baseline questionnaire [23–27]. Detailed information was obtained on sociodemographic and health behavior factors (education, marital status, employment status, occupational exposures, dietary intake, alcohol consumption, and tobacco use).

#### Cigarette smoking patterns and behavior

The survey contained a series of questions designed to identify cigarette smoking history. Respondents were asked if they had smoked greater than 100 cigarettes in their lifetime; those answering no were classified as nonsmokers. Those responding affirmatively were asked a series of follow up questions to determine their age when they started smoking and their smoking status (current vs. former) at the time of the survey. Those who smoked cigarettes during the past 30 days were classified as current smokers. Former smokers (not having smoked in the last 30 days) were asked the age at which they stopped smoking cigarettes. Intensity of smoking was measured as mean number of cigarettes smoked daily and pack-years of smoking, which was calculated from age started smoking, current age or age stopped smoking. All respondents, regardless of cigarette smoking status, were asked about their second-hand cigarette smoke exposure (lived with someone who smoked cigarettes daily inside the home) as children (up through age 15 years) and as adults.

#### Covariates

Demographic variables in the descriptive analysis included age as a three-level variable (21–44, 45–64, and 65 years and older); marital status as married/partnered or not; level of education dichotomized as high school graduate (or GED) or less versus greater than a high school education; and annual household income below $25,000 annually, $25,000-50,000, or greater than $50,000. Body mass index (BMI) was calculated as weight in pounds / height in inches^2^ (self-reported) multiplied by 703 and categorized as underweight (< 18.5), normal (18.5–24.9), overweight (25–29.9), and obese (30 or greater). Housing type was categorized as a single-family home, multi-unit housing, or mobile home.

### Statistical analysis

The REDCap database was stored and backed up on servers in the University of Kentucky DATAQUeST center. Data were exported via REDCap to SAS datasets. Frequency distributions of the demographic characteristics of our sample of respondents were calculated using SAS v. 9.4 [28]. Descriptive statistics were computed to determine characteristics of the population. Bivariate analyses using chi-square tests (for nominal variables) and t-tests (for interval variables) were conducted, and prevalence ratios (PRs) were estimated with appropriate 95% confidence intervals [29].

Descriptive statistics were computed to determine characteristics of the sample. We compared current smokers, former smokers, and nonsmokers using chi-square tests for associations among factors that may account for the differences in the smoking status. Respiratory health outcomes, including the prevalence of asthma, COPD, and black lung disease, were calculated for the sample overall and stratified by the smoking status categories. Age of initiation and intensity of smoking measured in mean cigarettes per day and pack years were examined by age and gender among current smokers. Bivariate analyses were performed to examine the associations between smoking status and the presence of established risk factors at baseline. Crude prevalence ratios (PRs) with appropriate 95% confidence intervals were estimated.

The primary outcome variable—current smoking—was sufficiently prevalent (> 10%) in our sample that we used a log binomial regression model to calculate the adjusted PRs and 95% confidence intervals using the PROC GENMOD procedure in SAS [30, 31] and generally followed the approach as described by Spiegelman [32]. The initial set of covariates included in the model were those identified from the literature as primary risk factors for current smoking and which demonstrated a *p* < 0.20 in bivariate analysis. For variables that may have been collinear, such as financial need and household income, only one variable was selected to reduce multicollinearity. Because financial need had fewer missing values than income, we selected the variable for the final model. Respiratory health variables were not included among the predictors. From this set, an initial full regression model was fit and then reduced (with sex included in the model) to a final model including variables for which p was < 0.05. Less than 1% of data were missing for key variables, so models were run omitting individuals with missing data.

## Results

From November 2015, to July 2017, a total of 4291 dwellings were enumerated within 30 HUCs in the study area. From 1459 eligible households contacted, 1190 individual participants (82%) were recruited into the study. Of those, 218 participants did not complete the survey due to refusal, loss to follow up, or death. Therefore, 972 individuals completed the survey.

Table 1 shows the characteristics of the Mountain Air Project participants by smoking status. Of the 972 participants, 58% were women, and the median age was 54.9 years (range: 21 to 96 years). Forty-six percent of participants reported annual household income below $24,999, including 26% reporting annual household income of less than $10,000. Fewer than half of participants (42%) had obtained education beyond high school. Nearly one fifth (19%) of participants identified as disabled.

**Table 1:** Characteristics of participants by smoking status: The Mountain Air Project, 2015–17 (*n* = 972)

Characteristic	Overall		Smoking Status						P
			Never		Former		Current		
	N	%	N	%	N	%	N	%	
Age									
< 45	290	29.8	118	40.8	34	11.8	137	47.4	<.01
45–64	444	45.7	196	44.2	102	23.0	145	32.7	
65 and >	238	24.5	105	44.3	94	39.7	38	16.0	
Gender									
Male	401	41.3	152	38.1	113	28.3	134	33.6	<.01
Female	571	58.7	267	46.8	117	20.5	186	32.6	
Marital Status									
Partnered	559	57.5	268	48.1	140	60.9	149	26.8	<.01
Not Partnered	413	42.5	151	36.7	90	39.1	171	41.5	
Education (*n* = 971)									
HS degree or less	558	57.6	186	33.3	149	26.7	223	40.0	<.01
> HS degree	410	42.4	233	56.8	81	19.8	96	23.4	
Employment									
Not employed	708	72.9	261	37.0	184	26.1	261	36.9	<.01
Employed part time	49	5.0	19	39.6	10	20.8	19	39.6	
Employed full time	215	22.1	139	64.7	36	16.7	40	18.6	
Annual household income (*n* = 735)									
< $10 k	252	34.3	63	25.2	45	18.0	142	56.8	<.01
$10 k-$24,999	199	27.1	82	41.2	47	23.6	70	35.2	
$25 k-$49,999	138	18.8	66	47.8	40	29.0	32	23.2	
$50 k and >	146	19.8	94	64.4	35	24.0	17	11.6	
Last 12 m any in household need prescription medication but couldn’t afford (*n* = 971)									
No	785	81.0	358	45.8	184	23.5	240	30.7	<.01
Yes	186	19.0	60	32.3	46	24.7	80	43.0	
Perceived financial status (*n* = 964)									
Struggle to make ends meet	416	43.1	131	31.6	83	20.0	201	48.4	<.01
Enough to get by	364	37.8	165	45.5	97	26.7	101	27.8	
More than enough	184	19.1	115	62.8	50	27.3	18	9.8	
BMI (*n* = 924)									
Underweight/Normal	235	25.4	81	34.6	48	20.5	105	44.9	<.01
Overweight	276	29.9	114	41.6	67	24.5	93	33.9	
Obese	413	44.7	196	47.5	108	26.2	109	26.4	
As adult ever live with smoker									
No	346	35.6	267	77.2	51	14.7	28	8.1	<.01
Yes	626	64.4	152	24.4	179	28.7	292	46.9	
< 16 years old live with smoker									
No	286	29.4	158	55.2	58	20.3	70	24.5	<.01
Yes	686	70.6	261	38.2	172	25.2	250	36.6	

Of those individuals under the age of 45, 47.4% were current smokers and another 11.8% were former smokers. Forty percent of participants with a high school degree or less education reported being current smokers, as did 56.8% of participants reporting an annual household income of less than $10,000. Of participants who reported struggling financially to make ends meet, 48.4% were current smokers. Although the proportion of current smokers among men and women was similar (33.6 and 32.6%, respectively), there were significantly more females who reported not smoking (46.8%), compared to males (38.1%).

To better understand the possible long-term implications of smoking patterns, Table 2 reports participants’ self-reported respiratory health by smoking status. Among participants who reported being diagnosed by a health care professional with emphysema, 53.3% reported being a current smoker. Similarly, among those who reported a diagnosis of COPD, 51.5% reported being a current smoker. Current smoking (25.8%) among those diagnosed with black lung disease was lower.

**Table 2:** Respiratory health by smoking status: the Mountain Air Project, 2015–17 (*n* = 972)

	Overall		Smoking Status						
			Never		Former		Current		P
	N	%	N	%	N	%	N	%	
Current Asthma									
No	862	88.7	375	43.7	206	24.0	278	32.4	.47
Yes	110	11.3	44	40.0	24	21.8	42	38.2	
Emphysema diagnosis:									
No	897	92.3	403	45.1	211	23.6	280	31.3	<.01
Yes	75	7.7	16	21.3	19	25.3	40	53.3	
COPD diagnosis:									
No	767	78.9	372	48.7	178	23.3	215	28.1	<.01
Yes	205	21.1	47	23.0	52	25.5	105	51.5	
Chronic bronchitis diagnosis:									
No	814	83.7	375	46.2	187	23.0	250	30.8	<.01
Yes	158	16.3	44	28.0	43	27.4	70	44.6	
Black lung disease diagnosis: (*n* = 970)									
No	872	89.9	378	43.5	197	22.7	295	33.9	.056
Yes	98	10.1	40	41.2	32	33.0	25	25.8	

To elucidate the history and characteristics of smoking in this sample, Table 3 displays the mean age at initiation and intensity of smoking among participants who reported being current smokers. As seen in this table, participants reported smoking just under a pack of cigarettes per day on average.

**Table 3:** Age at initiation and intensity of smoking among current smokers by sex and age (*n* = 551)

Variable	Mean age at initiation (yrs)	***P*** value	Mean # cigarettes per day	***P*** value	Mean pack years	***P*** value
**Sex**						
**Male**	16.93	0.60	19.20	0.18	33.92	0.01
**Female**	17.33		17.59		26.00	
**Age**						
**< 45**	16.21	0.07	17.24	0.31	16.30	<.0001
**45–64**	17.70		19.17		36.48	
**65+**	18.55		18.50		48.92	

Most of those currently smoking initiated smoking during their teenage years. Males reported starting smoking slightly earlier than females, and younger adults (< 45 years) reported initiating smoking earlier than older adults (65+ years), though none of these differences were statistically significant. Females and males reported smoking similarly high numbers of cigarettes per day. Likewise, individuals across age groups reported smoking similar elevated numbers of cigarettes per day. Men reported a significantly greater number of pack years compared with women (33.92 vs 26.00; *P* = 0.01).

Table 4 contains the unadjusted PRs for current smokers and former smokers, both compared to nonsmokers. Current smoking was less common among participants who were overweight (PR: 0.80, 95% confidence interval (CI): 0.65–0.97) and obese (PR:0.63, 95% CI: 0.52–0.77). In addition, for those who currently smoke ever living with an adult who was a smoker increased the prevalence of current smoking by nearly seven times (PR: 6.9, 95% CI: 4.8–9.9).

**Table 4:** Unadjusted Prevalence Ratios (PR): current vs. nonsmokers; former vs nonsmokers *n* = 972

Variable	Total	%	Current Smoker			Former Smoker		
			n	PR	95%CI	n	PR	95% CI
Age								
< 45	290	29.8	137	2.04	1.51–2.74	34	0.48	0.34–0.66
45–64	444	45.7	145	1.61	1.19–2.17	102	0.73	0.59–0.90
65 and >	238	24.5	38	Ref	–	94	Ref	–
Gender								
Male	401	41.3	134	Ref	–	113	Ref	–
Female	571	58.7	186	0.88	0.74–1.04	117	0.72	0.58–0.88
Education (*n* = 971)								
HS degree or less	558	57.6	223	1.87	1.54–2.26	149	1.72	1.38–2.15
> HS degree	410	42.3	96	Ref	–	81	Ref	–
Partner status								
Partnered	559	57.5	149	Ref	–	140	Ref	–
Not partnered	413	42.5	171	1.49	1.27–1.76	90	1.09	0.89–1.35
BMI (*n* = 924)								
Underweight/normal	235	25.4	105	Ref	–	48	Ref	–
Overweight	276	29.9	93	0.80	0.65–0.97	67	0.99	0.74–1.33
Obese	413	44.7	109	0.63	0.52–0.77	108	0.95	0.73–1.25
As adult ever live with smoker								
No	346	35.6	28	Ref	–	14.7	Ref	–
Yes	626	64.4	292	6.9	4.8–9.9	28.7	3.4	2.6–4.4
< 16 years old live with smoker								
No	286	29.4	70	Ref	–	58	Ref	–
Yes	686	70.6	250	1.6	1.3–2.0	172	1.5	1.2–1.9
Household income (*n* = 735)								
< $10,000	252	34.3	142	3.01	1.88–4.81	45	1.54	1.07–2.20
$10–$24,999	199	27.1	70	2.132	1.27–3.59	47	1.34	0.93–1.93
$25–$49,999	138	18.8	32	4.52	2.89–7.07	40	1.39	.96–2.02
$50,000 and >	146	19.8	17	Ref	–	35	Ref	–
Financial status (*n* = 964)								
Struggle to make ends meet	416	43.1	201	2.81	1.78–4.43	83		
Enough to get by	364	37.8	101	4.47	2.89–6.93	97	1.28	0.95–1.70
More than enough	184	19.1	18	Ref	–	50	Ref	–
Employment								
Not employed	708	72.9	261	2.24	1.47–3.40	184	1.68	0.94–2.99
Employed part time	49	5.0	19	2.24	1.68–2.98	10	2.01	1.47–2.74
Employed full time	215	22.1	40	Ref	–	36	Ref	–
Have dusty job (*n* = 875)								
No	529	60.5	153	Ref	–	115	Ref	–
Yes	345	39.5	126	1.41	1.18–1.67	101	1.52	1.23–1.87
Current asthma								
No	862	88.7	278	Ref	–	206	Ref	–
Yes	110	11.3	42	1.15	0.91–1.45	24	1.00	0.71–1.40
Ever Dx emphysema								
No	897	92.3	280	Ref	–	211	Ref	–
Yes	75	7.7	40	1.74	1.44–2.10	19	1.58	1.14–2.18
Ever Dx black lung/pneumonia (*n* = 970)								
No	872	89.9	295	Ref	–	197	Ref	–
Yes	98	10.1	25	0.88	0.64–1.21	32	1.30	0.98–1.72
Ever Dx chronic bronchitis								
No	814	83.7	250	Ref	–	187	Ref	–
Yes	158	16.3	70	1.54	1.29–1.83	43	1.49	1.17–1.89
Ever Dx COPD								
No	767	78.9	215	Ref	–	178	Ref	–
Yes	205	21.1	105	1.89	1.62–2.19	52	1.62	1.20–2.03

Table 5 provides the adjusted PRs for current smoking versus not smoking (referent). In this model, age younger than 65 years remained statistically significantly associated with current smoking, as did high school graduation or less education (PR:1.49, 95% CI: 1.23–1.81) and physician diagnosed depression (PR: 1.19, 95% CI: 1.03–1.37). Participants reporting less than enough finances for needs had more than three times the prevalence of current smoking compared to nonsmokers (PR: 3.15, 95% CI: 2.99–4.96).

**Table 5:** Adjusted prevalence ratios for current smokers vs. nonsmokers (*n* = 730)

	Current smoking (*n* = 730)		
	aPR	95% CI	*p*-value
Sex			
Female	0.99	0.86–1.13	0.84
Male	Ref.	–	–
Age			
21–44 years	1.55	1.16–2.06	.002
45–64 years	1.86	1.40–2.47	<.0001
Greater than or equal to 65 years	Ref.	–	–
Education			
High school graduation or less	1.49	1.23–1.81	<.0001
Education beyond High school	Ref.		
Financial need			
Less than enough	3.15	2.00–4.96	<.0001
Just enough	2.40	1.52–3.79	.0002
More than enough	Ref.		–
Physician diagnosed depression			
Yes	1.19	1.03–1.37	.021
No	Ref.		–

## Discussion

This central Appalachian population presents a unique smoking profile, relative to that of the broader United States population [33]. As of 2019, 14.0% of US adults smoke cigarettes [1]. Our unadjusted prevalence of 32.9% is nearly triple that goal. Furthermore, MAP participants reported smoking intensity higher than that reported among urban smokers [3]. It is unclear what is fostering this elevated smoking prevalence and intensity. It is plausible that historical reliance on tobacco for economic subsistence contributed to a high level of acceptability of smoking. Such acceptability and normative behavior may have de-stigmatized smoking and may encourage its widespread use in Appalachia. Finally, given the historical “tight knit” character of many Appalachian communities, it is possible that smoking could “spread” to families and friends [6]. It is well established that health behaviors such as smoking tends to reproduce, particularly among close relations [34].

Cigarette smoking is the leading cause of preventable morbidity and mortality in the US [33]. This study provides the most detailed description of extensive adult smoking in an Appalachian cohort to date. Approximately one third of adults in our study currently smoke, which is more than double the prevalence of U.S. adults (14.0%) according to the National Health Interview Survey [1]. While smoking in the U.S. has declined significantly over the last few decades [35], our findings suggest the opposite trend in this population. While Schoenberg and colleagues [12] estimated smoking prevalence among men in Appalachian Kentucky in 2010 as 30.9%, the prevalence of smoking among male participants in the present study (33.6%) suggests increasing rates of smoking. Our analyses revealed multiple factors associated with current smoking in this Appalachian population. We found, as others have, that lower education is associated with smoking. Participants who had attained at most a high school degree reported 1.4–2.2 times the prevalence of current smoking compared to those with education beyond high school. This pattern is consistent with state-level data indicating significantly higher smoking rates among adults with less than a high school education compared with adults with a college degree (38.9% vs 8.9%) [36]. Other studies also have found greater education to be associated with less smoking [37–39].

Younger age of smoking initiation was another factor associated with current smoking. With 87% of adult cigarette smokers across the US reporting having tried cigarette smoking by age 18, adolescence has been considered the peak time of tobacco use initiation [33]. Those participants who were age 45 years and younger reported earlier age of initiation relative to a previous study in rural Appalachia [40]. We noted earlier initiation of smoking among the younger age groups (16.2 years for < 45 age group), compared to 18.6 years for 65+ age group. Possible explanations for this finding include early smoking initiators over age 65 having already died or residence in an assisted living facility. In contrast with our findings, national data sources demonstrate a shift in peak age of smoking initiation from adolescence to young adulthood [41], indicated by higher initiation rates among young adults (6.3%) vs among adolescents (1.9%) [42].

Age of smoking initiation is a significant predictor of future smoking behavior, including heavy smoking, daily smoking and difficulty quitting smoking [43–45]. While our data indicate similar smoking prevalence between women and men, the earlier initiation of smoking among men (and associated greater number of pack years) provides an opportunity for targeted intervention. Gender-sensitive and gender-specific smoking prevention campaigns, including social and mass media campaigns, efforts to reduce children’s exposure to cigarettes at home, and school-based approaches to prevent tobacco use may be most effective if initiated earlier among males [46].

Our findings underscore the need for both smoking cessation and tobacco prevention initiatives in this rural, Appalachian population. Specifically, our development of a profile of current smokers allows for more precise tailoring, a promising approach that has been used in a diverse array of environments [47]. Since our data indicate elevated smoking rates among younger people, those with lower socioeconomic status, and those reporting depression, such groups warrant additional focus. Tailoring might include specific recruitment efforts and special programming; for example, “bundling” smoking cessation with mental health programming to address depression may support addressing multiple behavior change. Such multiple behavioral interventions, while more complex, have resulted in additive benefit [48]. Although smoking cessation programs exist in the Appalachian context [49], none of the programs are tailored or even targeted toward these personal characteristics. Furthermore, few of these programs leverage critical determinants of smoking cessation—social norms, peer support, and addressing logistical issues like affordability. For smoking prevention, the lower age at first initiation of smoking compared to national data point to the need for enhanced development and enforcement of policies, such as point of sale restrictions or increased taxes. A recent review highlighted counter-industry marketing, denormalization campaigns, smoke-free policies and cigarette tax increases as effective in deterring smoking initiation among young adults [46]. Raising the minimum age of legal access has also been associated with reduced smoking among adolescents [50]. The federal Tobacco 21 law, passed in 2019, superseded the Kentucky minimum age of 18 for sale of tobacco products, raising the minimum age from 18 to 21 [51]. This provides a natural experiment to monitor age at smoking initiation.

In addition to prevention initiatives, our findings point to the need for greater support of cessation efforts among individuals with respiratory diseases, given high prevalence of smoking among our participants with a medical diagnosis of COPD (51.5%) and emphysema (53.3%). Our findings corroborate those of previous researchers documenting high prevalence of smoking among individuals with COPD and asthma [52]. A recent report from the Centers for Disease Control and Prevention documented a national age-adjusted prevalence of COPD among current cigarette smokers to be 15.2% [53]. The same report noted positive associations between state-wide prevalence of COPD and state-wide prevalence of current smoking, across individual smoking statuses (current, former, and never) [53].

The persistently high rate and intensity of smoking in Appalachian Kentucky represents a confluence of missed opportunities in public policy and poverty resulting from a regional economy historically pervaded by extractive industries such as coal mining and natural gas drilling. While tobacco has lost its status as the state’s leading agricultural product, the industry continues to play an outsized role in influencing Kentucky’s health disparities. For decades, Kentucky maintained the second lowest state tax on cigarettes in the nation, 3 cents per pack. This tax was not increased until 2005, when the tax rose to 30 cents per pack. In 2018, the cigarette tax reached its current state rate of $1.10 per pack, still well below the national median of $1.70 [54]. During the 2018 Kentucky General Assembly, the tobacco giant Altria spent a record-breaking $379,760 to successfully lobby against a proposed one-dollar per pack cigarette tax, spending twice as much as the next highest industry [55]. To date, there is no state-wide ban on indoor smoking. A number of local jurisdictions have adopted their own restrictions but the most comprehensive of these only protect 30% of the state’s population. Further, indoor smoking bans are more likely to cover urban rather than rural populations [56]. Neither has the state’s effort to prevent tobacco addiction been robust. Kentucky received $507.3 million from the tobacco settlement in 2019 but only 0.75% of these funds were used for tobacco prevention efforts [57].

As noted earlier, lower educational attainment is a strong, independent predictor of smoking [12]. Given this, equitable investment in public education might be considered an “upstream” strategy for reducing the prevalence of tobacco use. However, public education in Kentucky has historically been underfunded because of its ties to local property taxes. This is particularly true for schools in lower resourced communities, including Appalachian Kentucky. A study of landownership patterns in 80 Appalachian counties [58] found that land and mineral resources were largely held by corporate absentee owners who benefitted from a pattern of tax exemptions and undervaluation of that resulted in restricted county property tax bases [59]. School districts in the lowest quintile of funding are largely concentrated in Appalachian Kentucky and include our study counties Letcher and Harlan [60]. The experience of Appalachian Kentucky suggests that the schemes of public education that rely primarily on local wealth inevitably disadvantage education in rural and impoverished regions, and alternative funding strategies may serve as a mechanism for achieving greater health and educational equity.

### Limitations

There are several limitations of our study. Smoking status was self-reported and not biochemically validated. This may not be a major limitation, however, as smoking status is a valid proxy for serum cotinine levels in multiple studies including a nationally representative study [61–63]. Other variables, including BMI and chronic conditions, similarly were self-reported. Additionally, our sampling approach prioritized enrolling those who reported respiratory illness. This sampling approach was designed to meet multiple objectives in the study including increasing the efficiency for the epidemiologic analysis by augmenting slightly those with health outcome and collecting baseline data among asthmatics for a later planned intervention. These objectives were part of the community engaged design. While this may lead to an upward bias in the prevalence estimates for the respiratory outcomes and potentially a (likely) upward bias for smoking status, it would have no impact on the prevalence ratios in the log binomial analysis. These estimates of association would still be unbiased. A final limitation involves a lack of focus on poly tobacco use (concurrent use of two or more tobacco products). Research has established that Appalachian residents have elevated rates of poly tobacco use [64], which may complicate smoking cessation. Strengths of our study include a high participation rate and the similarity between our sample and the demographics of the local population [20].

## Conclusions

Although nationally smoking rates are at an all-time low, some populations, including those residing in rural Central Appalachia, have not experienced such steep decreases in tobacco use. As a result, rural Appalachian residents continue to suffer tremendous (and preventable) health and economic burdens from smoking. Our findings underscore the need for tobacco prevention initiatives in this rural, Appalachian population. In particular, the higher prevalence at lower age group and lower age at first initiation compared to national data point to the need for enhanced tobacco control policies, such as taxes, smoke-free policies, and regulation of marketing practices. Historically, such policies and practices have not been widely implemented in rural communities [65, 66].

## Data Availability

The datasets generated and/or analysed during the current study are not publicly available since they are embargoed for a 2-year time period while investigators are writing the initial papers but are available from the corresponding author on reasonable request.

## Abbreviations

US: United States
COPD: Chronic obstructive pulmonary disease
MAP: Mountain Air Project
UK: University of Kentucky
HUC: Region defined by a hydrologic unit code
CHW: Community health worker
BMI: Body mass index
PR: Prevalence ratio
CI: Confidence interval
